# Bioverm^®^ in the Control of Nematodes in Beef Cattle Raised in the Central-West Region of Brazil

**DOI:** 10.3390/pathogens10050548

**Published:** 2021-05-01

**Authors:** Lucineide da Silva Santos Castelo Branco de Oliveira, Felipe Guerra Santos Dias, Andréia Lima Tomé Melo, Lorendane Millena de Carvalho, Edir Nepomuceno Silva, Jackson Victor de Araújo

**Affiliations:** 1 Faculty of Veterinary Medicine, Universidade de Cuiabá, Cuiabá 78557-287, MT, Brazil; lucineidebenvenuti@gmail.com (L.d.S.S.C.B.d.O.); felipeguerra7@hotmail.com (F.G.S.D.); andreia.melo@kroton.com.br (A.L.T.M.); 2 Department of Veterinary, Universidade Federal de Viçosa, Viçosa 36570-900, MG, Brazil; jvictor@ufv.br; 3 Faculty of Food Engineering, Universidade de Campinas, Campinas 13083-970, SP, Brazil; edir.nepo@ghenvet.com

**Keywords:** cattle farming, nematophagous fungi, *Duddingtonia flagrans*, gastrointestinal nematodes

## Abstract

*Cooperia*, *Haemonchus* and *Oesophagostomum* are the genera of gastrointestinal parasitic nematodes most prevalent in cattle and constitute a serious problem in cattle breeding due to the impact they have on meat and milk production and the high costs of control measures. The objective of the present work was to evaluate the efficiency of Bioverm^®^ (*Duddingtonia flagrans*) in the control of gastrointestinal parasitism of young cattle raised in the field, in the Central-West region of Brazil. The experiment was conducted on a farm located in the municipality of Jangada, MT, where 18 cattle, Nelore and Aberdeen Angus breeds, aged six to ten months, were randomly divided into two groups (treated group and control group) and distributed in paddocks of *Brachiaria decumbens*, naturally infested by larvae of gastrointestinal nematodes. The animals in the treated group received 1g of Bioverm^®^ for each 10 kg of body weight, administered daily with commercial feed, throughout a period of six months. In the control group, each animal received 1 g of rice bran for each 10 kg of body weight, without Bioverm^®^, added to the feed. Stool and pasture samples were collected every two weeks. The treated group showed a significant reduction (*p* < 0.05) in values of eggs per gram of feces (EPG) and a significant gain of body weight (*p* < 0.05) when compared to the control group. The fungal formulation Bioverm^®^ was effective in pasture decontamination and consequently in reducing the occurrence of reinfection by nematodes. The animals treated with Bioverm^®^ showed a lower parasitic load and greater weight gain.

## 1. Introduction

Gastrointestinal nematodes in livestock reduce productivity which causes economic losses [1]. The helminths of the genera *Cooperia*, *Haemonchus* and *Oesophagostomum* comprise an important group of disease-causing parasites in grazing ruminants [2]. The most used strategy to control this problem has been the use of chemical drugs, which can present some disadvantages, such as ecotoxicity [3] and the development of populations of multidrug-resistant parasites against most anthelmintic classes [4,5].

Thus, biological control using nematophagous fungi has been tested in the field [6], by breaking the life cycle, capturing infective larval phases before migrating from the fecal pats to pasture and thereby decreasing relapses of helminth infections that cause damage to livestock and, in particular, cattle [7,8,9,10].

The fungus *D. flagrans* has high conidia production and produces chlamydospores. This species is the most commonly evaluated and most efficient in the control of nematodes in ruminants [11,12,13]. These characteristics are important in a nematophagous fungus for it to have greater success as a nematode controller. Fungi that have high production of conidia and chlamydospores can disperse and colonize the environment [14].

The objective of this work was to evaluate the effectiveness of Bioverm^®^ (*D. flagrans*) in the control of gastrointestinal parasitism in young cattle raised in the field.

## 2. Results

### 2.1. Anthelmintic Efficacy

Daily administration of Bioverm^®^ proved to be effective in reducing EPG throughout the experiment. The daily use of 1g for every 10 kg of body weight caused the greatest significant reduction in EPG (87.5%) observed on day 90.

**Figure 1:**
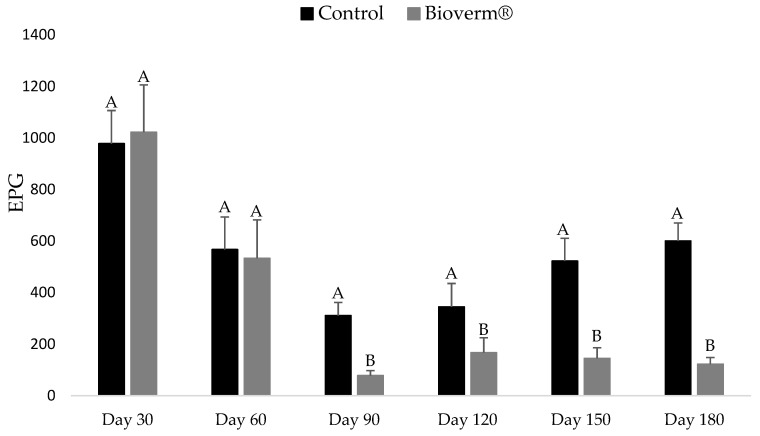
Means and standard deviations of eggs per gram of feces (EPG) of cattle in the group treated with Bioverm^®^ and control (without fungus), for 180 days in the state of Mato Grosso, Brazil, from February to August 2020. Values with the same letters are statistically similar using Tukey’s test (*p* < 0.05).

In the first month of treatment, the low number of EPG was probably due to the anthelmintic treatment administered previously. A significant difference (*p* < 0.05) was observed in the animals’ EPG from day 90 after the start of Bioverm^®^ administration, and this difference was maintained throughout the remainder of the experiment. The reduction of EPG in the treated group reached 87.5% when compared to the control group (Figure 1). On the other hand, there was a significant drop in the EPG of both groups on the 90th day. This is possibly due to the reduction in the percentage of larvae in the pasture between the months of May and June, leading the animals to ingest fewer larvae and, therefore, eliminate fewer eggs.

### 2.2. Climate Data

**Figure 2:**
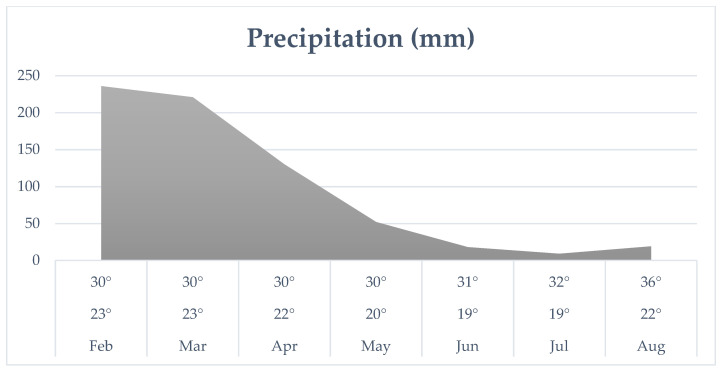
Average monthly maximum, mean and minimum temperatures (°C) and monthly rainfall (mm^3^) recorded from February to August 2020, Jangada, Mato Grosso, Brazil.

Figure 2 shows the maximum, average and minimum temperatures, as well as the average monthly precipitation, during the experimental period. In general, it was found that temperatures and precipitation influence the environmental parasitic load.

### 2.3. Gastrointestinal Nematodes 

In coprocultures, it was observed that the most prevalent genus of gastrointestinal parasitic nematode was Haemonchus spp., with values between 56 and 100% throughout the experiment. The second most prevalent genus was Cooperia spp., with a prevalence of 20 to 31%. The least prevalent genus was Oesophagostomum spp., with a 7 to 14% prevalence. Only one exception to this prevalence occurred during the experiment, in the control group, on day 180, which presented only 29% Haemonchus spp., 57% Cooperia spp. and 14% Oesophagostomum spp. (Table 1).

### 2.4. Animal Weight Gain

**Figure 3:**
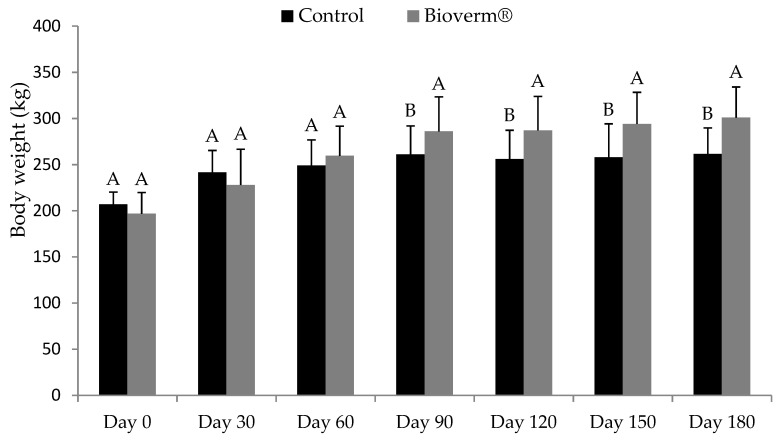
Means and standard deviations of body weight (kg) of cattle in the group treated with Bioverm^®^ at a daily dose of 1 g per kilogram of body weight and control, without fungus, for 180 days in the state of Mato Grosso, Brazil, in the period of February to August 2020. Values with the same letters are statistically similar by Tukey’s test (*p* < 0.05).

During the weight evaluations of the animals, greater weight gain (*p* < 0.05) was observed in the animals that received Bioverm^®^ compared to the animals in the control group, with a significant difference from day 90 (Figure 3). At the end of the experiment, the treated animals had an average weight of 300 kg, with an average weight gain of 107 kg, while the animals in the control group had a final average weight of 261 kg, with an average weight gain of 54.3 kg (Figure 3).

### 2.5. Infective Larvae per Kilogram of Dry Matter

**Figure 4:**
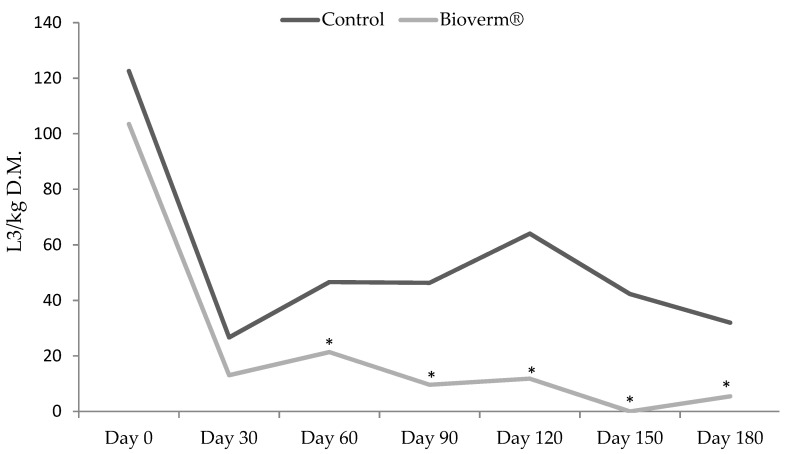
Number of infective larvae/kg of dry matter (L3/kg DM) recovered from pasture fed by cattle in the group treated with Bioverm^®^ at a daily dosage of 1 g per kilogram of body weight and control, without fungus, for 180 days, in the municipality of Jangada, Mato Grosso, Brazil from February to August 2020. Asterisks on the same day indicate a significant difference in Tukey’s test (*p* < 0.05).

In the paddock where pasture was consumed by the animals in the treated group, the values of infective larvae per kilogram of dry matter (L3/kg DM) gradually decreased throughout the experiment (*p* < 0.05), while what was consumed by the control group without fungus showed large numbers of larvae recovered throughout the experiment, as shown in Figure 4. At the end of the experiment, the reduction of larvae recovered from the pasture of the treated group compared to the control group was 100% on day 150 and 82.9% on day 180.

On the other hand, on 30 March, the percentage of reduction fell, that is, the number of larvae recovered from pasture decreased in both groups, as shown in Figure 4, probably due to the increase in the rainfall index recorded at that timeframe. In January, February and March 2020 in the city of Jangada, there was more precipitation and increased temperature.

## 3. Discussion

The cattle in the group treated with Bioverm^®^ exhibited a reduction in eggs per gram of feces (EPG) of 87.5% in the present study. According to Raynauld and Gruner [15], EPG counts allow for monitoring the levels of infection in animals and pasture infestation by parasitic gastrointestinal nematodes. It should also be considered that this reduction occurred after three months of treatment with fungus. Several studies using *D. flagrans* fungus in horses and ruminants have also reported lower monthly mean EPG counts among treated animals compared to control groups in different locations and climatic conditions: for sheep in the USA [16]; for goats in France [17]; for horses in Brazil [7]; for cattle in southeastern Brazil [18].

Vilela et al. [19] administered pellets containing the fungi *D. flagrans* and *Monacrosporium thaumasium* to sheep and demonstrated that this treatment was able to control gastrointestinal helminths in the semiarid region of Northeast Brazil. These findings are in agreement with the results of the present study, confirming the action of the fungus against the infectious forms of the fecal environment and the consequent decrease in EPG.

The coprocultures revealed the occurrence of nematodes of the genera *Haemonchus*, *Cooperia* and *Oesophagostomum* during the experimental period (February 2020–August 2020). This result corroborates [20], who reported *Cooperia* spp. and *Haemonchus* spp. as the most prevalent genera of bovine parasites in Brazil, followed by *Oesophagostomum* spp. According to [21], the importance of these parasites for livestock production is directly related to the reduction in weight gain, production losses and the high resistance that these gastrointestinal parasitic nematodes have developed to anthelmintics. In 2004, Araújo et al. [22] demonstrated that the fungus *D. flagrans* is not selective for a specific species of parasite. Climatic conditions may have influenced the variations observed in the proportions of nematode genera in the present study

Elevated values of L3 (infective larvae) were observed on day 30 in both groups (average of 977 and 1022 for the groups treated with Bioverm^®^ and control, respectively), which can be explained by the short period for the colonization of fungus. The constant administration of fungi releases more and more chlamydospores into the environment, leading to an increase in the dispersion of fungi and colonization throughout the pasture. This is attributed to the increase in the predatory capacity of the fungus in the environment, which gradually reduces the EPG of the animals. Several studies evaluating *D. flagrans* in the control of ruminant worms have also shown that efficacy occurs from 60 days after the start of administration [10,18,23,24]. 

Likewise, Araújo et al. [22] reported that the combination of *D. flagrans* and *M. thaumasium* resulted in a 90.7% reduction in the number of larvae in coprocultures. Burke et al. [25] demonstrate in field experiments with *D. flagrans* that this fungus can reduce by more than 90% the infectious larvae present in the feces. The use of Bioverm^®^ in the present study showed action on the free-living form of the nematode larvae, which reduced the contamination of the pastures.

It is important to report that temperature, relative humidity and precipitation promoted the development of free-living stages and migration to forage. The climate is directly related to the ability of fungi to capture nematodes and it is important to know the optimum growth temperature of each species of fungus [26]. Other researchers also observed that temperatures in the range of 20 °C to 33 °C influenced the percentage of larvae trapped in different species of fungi [27,28].

Climatic conditions of the Central-West region of Brazil may be related to the results of the present study. Castro et al. [29] reported that *Arthrobotrys robusta* was able to capture larvae at temperatures ranging from 25 °C to 28 °C and *M. thaumasium* was not affected by temperatures from 25 °C to 30 °C, which confirms that the influence of temperature depends on the species or genus of nematode. 

The result of the present study is similar to previous studies and suggests that the efficiency with which larvae are controlled by nematophagous fungi depends on the fungus species and its suitability for specific conditions of temperature and rainfall. These results can support and explain the sudden drop in the number of larvae recovered from pasture in both groups (month 3 of the study). In this study, the temperature in the region varied from 30 °C to 36 °C, and the authors observed that the high temperature did not affect the performance of the Bioverm^®^ product. Furthermore, Van Dijk et al. [30] claimed that levels of infection are higher during the rainy season. Higher humidity can favor the development of parasitic free-living stages and the migration of L3 from feces to adjacent pastures.

The reduction in fecal egg count was inversely proportional to weight gain, similar to the work by Luns et al. [8], who evaluated the combination of *D. flagrans*, *M. thaumasium* and *A. robusta* in cattle for six months. The development of the fungus in the pasture has already been evaluated in several studies, significantly reducing the amount of L3 in the pasture and consequently the reinfection of the animals [8,19].

The animals that received Bioverm^®^ obtained rates of weight gain almost twice as high as the animals in the control group (average of 107 kg in the treated group, compared to an average of 54.3 kg in the control group), leading to significant economic gains, both by increasing these rates and reducing the costs of deworming. The costs of deworming are very high and, hence, most commercial farms do not monitor the economic losses due to deworming [2]. 

The results obtained in the present study demonstrated that the use of Bioverm^®^ was efficient in the control of bovine gastrointestinal nematodes, and may be an alternative to reduce the damage related to parasitic diseases in livestock.

## 4. Materials and Methods

### 4.1. Products

The commercial product evaluated was Bioverm^®^ (GHENVET Saúde Animal, Paulínia, SP, Brazil), which is composed of rice bran containing 10^5^ chlamydospores of the fungus *D. flagrans* per gram.

### 4.2. Experimental Assay In Vivo

The experimental assay was conducted on a farm located in the municipality of Jangada, state of Mato Grosso, Brazil, latitude: −15.2413, longitude: −56.4864, 15°14′29″ south, 56°29′11″ west, tropical climate with a dry season, maximum annual average temperature of 26 °C and a minimum temperature of 19 °C and with an average annual precipitation of 500 mm.

Eighteen Nelore and Aberdeen Angus cattle, aged six to ten months, with an average initial weight of 180 kg, were previously treated with the anthelmintic ivermectin 1% at a dose of 1 mL/50 kg of body weight and albendazole suspension in a dose of 1 mL/20 kg of body weight. Twenty-one days after the anthelmintic treatment and with confirmation of the absence of nematode eggs in the feces, the animals were randomly divided into two groups with nine animals each and distributed in two paddocks with two hectares each of *Brachiaria decumbens,* naturally infested with helminth larvae, with a pasture history of young animals.

In the treated group, each animal received one gram of Bioverm^®^ for each 10 kg of body weight, administered daily together with commercial feed. In the control group, each animal received 1 g of rice bran for each 10 kg of animal live weight, without Bioverm^®^ added to the feed. 

The experiment lasted seven months (February to August 2020). The entire proposed methodology was in accordance with the guidelines of the World Association for the Advancement of Veterinary Parasitology (WAAVP), following the guidelines for assessing the effectiveness of anthelmintics in ruminants (cattle and sheep) described by Powers et al. [31] and the second edition of the guidelines to evaluate the effectiveness of anthelmintics in ruminants (cattle, sheep and goats) cited by Wood et al. [32].

### 4.3. Collection and Processing of Feces

Every 2 weeks, after the introduction of the animals in the pastures, stool samples from all animals in each group were collected directly from the rectal ampoule. In these samples, egg counts per gram of feces (EPG) were determined by the method of [33], modified by Lima [34].

Additionally, every 14 days, coprocultures were produced with 20 g of feces mixed with vermiculite and put into an environmental chamber, at 26 °C for fifteen days, to obtain infective larvae of parasitic gastrointestinal nematodes, which were subsequently identified according to the criteria established by Keith [35].

### 4.4. Pasture Samples

Every 14 days, two pasture samples (0–20 and 20–40 cm away from the fecal pats) were collected from the paddocks of the treated and control groups from six alternate points, according to Raynauld and Gruner [15]. Samples of 500 g of pasture were weighed, and the infectious larvae (L3) of bovine gastrointestinal nematodes were recovered, following the methodology described by [34]. Subsequently, the sediment was examined under a light microscope and the larvae were counted and identified according to the criteria established by Keith [35].

The samples of pasture were placed in an oven at 100 °C for three days to obtain dry matter. The data obtained were transformed into the number of larvae per kilogram (kg) of dry matter.

### 4.5. Animal Weight Gain

The animals’ weight was estimated monthly by a digital scale, from the beginning to the end of the experiment.

### 4.6. Climate Data

Weather data referring to the average monthly minimum, average and maximum temperatures, as well as monthly precipitation, were recorded in a weather station, Climatempo data (https://www.climatempo.com.br/), in the city of Jangada, in the state of Mato Grosso, Brazil.

### 4.7. Statistical Analysis

The variables were submitted to tests of normality and homoscedasticity and then to analysis of variance. The means of the variables were compared using Tukey’s test, adopting a 5% level of probability.

## 5. Conclusions

The fungus *Duddingtonia flagrans* in the commercial product Bioverm^®^ was proved to be viable in the environmental control of gastrointestinal nematode larvae, causing a significant reduction in the EPG of the animals, increasing their weight gain and significantly reducing the contamination of the pasture by infective nematode larvae.

## Figures and Tables

**Table 1:** Percentage of infective larvae of *Haemonchus* spp. (H), *Cooperia* spp. (C) and *Oesophagostomum* sp.(O) in the group treated with Bioverm^®^ at a daily dosage of 1 g per kilogram of body weight and control, without fungus, recovered from fecal cultures of cattle for 180 days in the state of Mato Grosso, Brazil, from February to August 2020.

Groups		Day 0	Day 30	Day 60	Day 90	Day 120	Day 150	Day 180
Control	H	63	100	64	60	72	82	29
	C	30	-	27	29	20	42	57
	O	7	-	9	13	8	11	14
Bioverm^®^	H	56	-	100	62	73	48	100
	C	31	-	-	27	18	20	-
	O	13	-	-	7	8	9	-

## Data Availability

The study did not report any data.
